# Telomerase as a Target for Therapeutic Cancer Vaccines and Considerations for Optimizing Their Clinical Potential

**DOI:** 10.3389/fimmu.2021.682492

**Published:** 2021-07-05

**Authors:** Espen Basmo Ellingsen, Sara M. Mangsbo, Eivind Hovig, Gustav Gaudernack

**Affiliations:** ^1^ Department of Tumor Biology, Institute for Cancer Research, The Norwegian Radium Hospital, Oslo, Norway; ^2^ Faculty of Medicine, University of Oslo, Oslo, Norway; ^3^ Research and Development, Ultimovacs ASA, Oslo, Norway; ^4^ Research and Development, Ultimovacs AB, Uppsala, Sweden; ^5^ Department of Pharmaceutical Biosciences, Science for Life Laboratory, Uppsala University, Uppsala, Sweden; ^6^ Centre for Bioinformatics, Department of Informatics, University of Oslo, Oslo, Norway

**Keywords:** cancer, telomerase, immunotherapy, cancer vaccine, hTERT, melanoma, immune response, immuno-oncology

## Abstract

Telomerase-based therapeutic cancer vaccines (TCVs) have been under clinical investigation for the past two decades. Despite past failures, TCVs have gained renewed enthusiasm for their potential to improve the efficacy of checkpoint inhibition. Telomerase stands as an attractive target for TCVs due to its almost universal presence in cancer and its essential function promoting tumor growth. Herein, we review tumor telomerase biology that may affect the efficacy of therapeutic vaccination and provide insights on optimal vaccine design and treatment combinations. Tumor types possessing mechanisms of increased telomerase expression combined with an immune permissive tumor microenvironment are expected to increase the therapeutic potential of telomerase-targeting cancer vaccines. Regardless, rational treatment combinations, such as checkpoint inhibitors, are likely necessary to bring out the true clinical potential of TCVs.

## Introduction

Preventive vaccines against infectious agents have been one of the major advancements in medical history. However, the transfer of this technology to the treatment of cancer has for several reasons proven to be a difficult task. Multiple therapeutic cancer vaccines (TCVs) have been evaluated in clinical trials since the 1990s, often inducing vaccine-specific immune responses, but rarely translating to clinical efficacy (1, 2). Nevertheless, the recent advances in immunotherapy have rekindled the interest in TCVs, exemplified by an increase from 612 to 855 cancer vaccine candidates in the overall immuno-oncology drug pipeline from 2017 to 2020 (3).

The advent of checkpoint inhibitors (CPIs) has provided significant improvement in survival outcomes for patients with various cancer types, most notably in malignant melanoma (4, 5). CPIs disrupt intrinsic and tumor-induced suppressor mechanisms restricting a spontaneous anti-tumor immune response, as evidenced by associations between tumor PD-L1 expression, tumor-infiltrating lymphocytes (TILs), tumor mutational burden (TMB), neoantigen load, and response to therapy (6–9). Although many patients experience remarkable durable clinical responses to CPIs, most patients eventually progress. A lack of response to CPIs is believed to be caused by either an insufficient spontaneously primed immune response against tumor antigens, an immunosuppressive tumor microenvironment (TME), or a combination of the two (10). These insights into the balance between the immune system and the tumor suggest TCVs as a logical next step to improve clinical outcomes by strengthening the immune response against the tumor and possibly overcoming TME immunosuppression. Moreover, there are good reasons to believe that the immune checkpoint molecules contributed to the earlier failure of TCVs in the clinic (11). Combining CPIs with TCVs may allow unchecked expansion and function of vaccine-induced T cells both in tumor-draining lymph nodes and in the tumor, thereby achieving superior anti-tumor immune responses, balancing the immune system in favor of tumor control.

An essential property of a TCV is the antigen it targets. Tumor-associated antigens (TAAs) are antigens that are over-expressed by the tumor and preferably have a restricted expression pattern in healthy tissues. Several TAAs have been the target of TCVs and can be divided into three broad categories: 1) germline antigens (e.g., MAGE and NY-ESO-1) (12), 2) cell lineage antigens (e.g., gp100, MART-1, PSA/PAP/PMSA) (2, 13–16), and 3) differentially expressed antigens (e.g., telomerase and Her2) (17, 18). A challenge with targeting endogenous antigens is the possibility of central tolerance, where self-reactive high-affinity T cell clones are deleted through negative selection in the thymus and are thus absent or present in low numbers with low affinity in the T cell repertoire (19). Another challenge is the risk for on-target off-tumor autoimmunity, where vaccination induces an immune response to healthy tissues expressing the antigen. One TCV strategy to circumvent these challenges is to vaccinate against predicted tumor neoantigens. Tumor-specific somatic mutations may give rise to aberrant peptides presented as T cell targetable neoantigens on the cancer cell surface in the context of an MHC molecule (20, 21). Though neoantigens are seemingly attractive targets for vaccination, subclonal expression of neoantigens due to substantial intratumoral heterogeneity may provide resistance and escape mechanisms for the tumor (9). Furthermore, such personalized TCVs require comprehensive logistics and subsequent delay in the onset of treatment.

In this review, we will discuss cancer vaccines targeting the differentially expressed TAA telomerase reverse transcriptase (hTERT), offering an “off-the-shelf” alternative to personalized TCVs. Telomerase is almost ubiquitously expressed in cancer, and TCVs targeting hTERT is thus an attractive approach to achieve T cell infiltration and epitope-spreading. We will focus on tumor biological considerations, immunologically rational indications and treatment combinations to optimize for clinical efficacy from therapeutic vaccination against telomerase. Furthermore, we will provide insights into possible causes of failure in previous clinical trials and an update on ongoing studies.

## Telomerase as a Target for Therapeutic Vaccination

### An Almost Universal Cancer Antigen

#### Telomeres and Telomerase

The 3’ ends of the chromosomes consist of a repeating sequence of nucleotides, TTAGGG, termed telomeres. The telomeres serve to protect the chromosomal ends from inducing DNA damage responses, which would otherwise be activated upon breaks in the double-stranded DNA. Since DNA polymerases cannot replicate the DNA ends, the telomeres are progressively shortened with approximately 50 bps with each cell division (22). This progressive shortening of the telomeres ultimately leads to telomere crisis and chromosomal instability and, consequently, a limited number of cell divisions that can occur before the cell enters senescence or apoptosis (23). This replicative cellular senescence phenomenon led to the identification of the Hayflick Limit, defined as the maximum number of cell divisions that can occur in a somatic cell (24). The reverse transcriptase enzyme component of the telomerase complex (hTERT) can be activated in specific cell types, leading to replication of the telomeric DNA and thereby increasing the proliferative potential of the cell (23).

In somatic cells, telomerase is restricted to certain rapidly proliferating tissues, such as the intestinal epithelium, premenopausal endometrium, the testis, and tissues containing a high population of activated lymphocytes, such as secondary lymphoid organs (25). Telomerase is also expressed by stem cells, and their telomerase activity is closely related to the proliferation rate, explaining the relatively low activity in adult stem cells compared to embryonic or cancer cells (26).

#### Telomerase in Cancer

**Figure 1:**
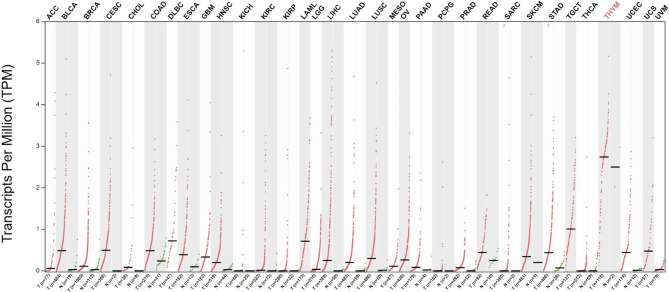
Differential expression of hTERT in tumors vs. adjacent normal tissues. Red dots indicate primary tumor, and green adjacent healthy tissue. Data gathered from The Cancer Genome Atlas through GEPIA (32). Q-value cut-off was set to 0.01. ACC, Adrenocortical carcinoma; BLCA, Bladder Urothelial Carcinoma; BRCA, Breast invasive carcinoma; CESC, Cervical squamous cell carcinoma and endocervical adenocarcinoma; CHOL, Cholangiocarcinoma; COAD, Colon adenocarcinoma; DLBC, Lymphoid Neoplasm Diffuse Large B-cell Lymphoma; ESCA, Esophageal carcinoma; Glioblastoma multiforme GBM, Head and Neck squamous cell carcinoma HNSC, KICH, Kidney Chromophobe; KIRC, Kidney renal clear cell carcinoma; KIRP, Kidney renal papillary cell carcinoma; LAML, Acute Myeloid Leukemia; LGG, Brain Lower Grade Glioma; LIHC, Liver hepatocellular carcinoma; LUAD, Lung adenocarcinoma; LUSC, MESO, Lung squamous cell carcinoma; Mesothelioma; OV, Ovarian serous cystadenocarcinoma; PAAD, Pancreatic adenocarcinoma; PCPG, Pheochromocytoma and Paraganglioma; PRAD, Prostate adenocarcinoma; READ, Rectum adenocarcinoma; SARC, Sarcoma; SKCM, Skin Cutaneous Melanoma; STAD, Stomach adenocarcinoma; TGCT, Testicular Germ Cell Tumors; THCA, Thyroid carcinoma; THYM, Thymoma; UCEC, Uterine Corpus Endometrial Carcinoma; UCS, Uterine Carcinosarcoma; UVM, Uveal Melanoma.

Telomerase has been extensively studied in cancer, and telomerase activity has been documented in >90% of all cancers (27, 28). Telomerase activation is a major cell immortalization mechanism and is implicated as an essential step in carcinogenesis (29). Through telomerase activation, cancer cells acquire the ability of unlimited proliferation. Telomerase activity is also linked to epithelial-to-mesenchymal transition and cancer stemness, providing cancer cells with metastatic potential (30, 31). Telomerase is expressed in most tumor types across all stages of development and is thus an attractive target for therapeutic vaccination (**Figure 1**). To restrict possibilities of resistance mutations to develop, epitopes within the essential hTERT component of the telomerase complex are commonly used as antigens for TCVs, as loss of hTERT would abolish tumor growth. Moreover, due to its ubiquitous expression, hTERT serves as a cancer antigen being independent of clonal diversity within a tumor. Tumor telomerase activity is considered a negative prognostic factor for several cancers (33–37), while spontaneous anti-hTERT CD4+ immune responses have been identified as a positive prognostic factor in non-small cell lung cancer (NSCLC) (38), substantiating both the natural immunogenicity of hTERT and its relevance as a target for TCVs.

### Telomerase Vaccination

TCVs aim to induce T cells that target a tumor antigen leading to improved anti-tumor immune responses and, ultimately, cancer cell death. As recently reviewed, telomerase vaccination has been evaluated across 34 clinical trials spanning almost two decades (39). However, there have been no positive late-phase studies, and as such, there is an obvious need for improvement, either in vaccine design (including sequence choice, formulation, and delivery), selection of indications, or treatment combination strategies. Optimal hTERT targeting TCVs should be designed to effectively induce the appropriate immune response phenotype, which can be further augmented through rational therapeutic combination strategies.

#### The Phenotype of the Induced Immune Response

**Figure 2:**
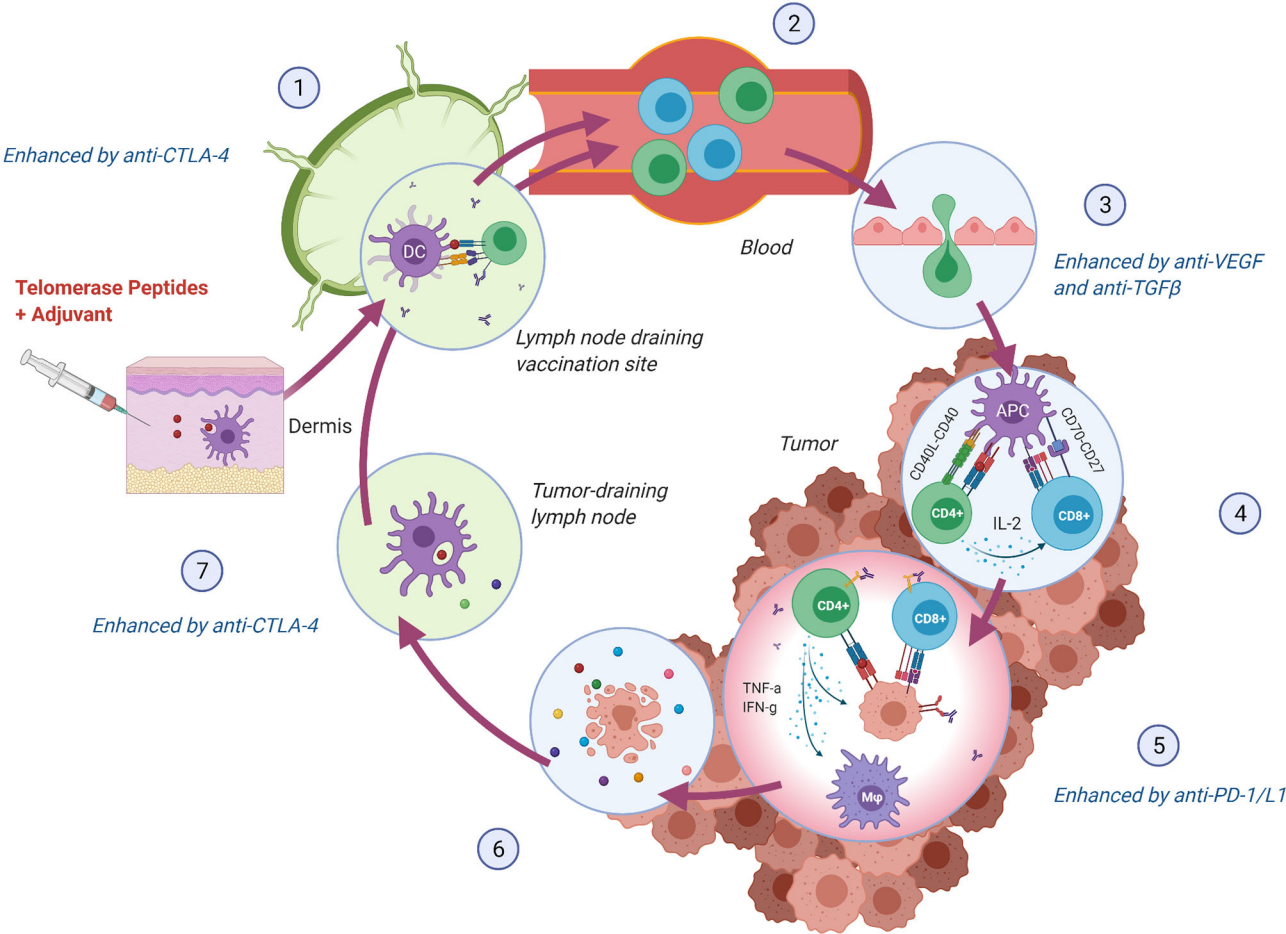
hTERT specific CD4+ Th1 cells support crucial steps in the Cancer Immunity Cycle (51). (1) Vaccine peptides are presented to naïve T cells by DCs in the lymph node draining the vaccination site. Anti-CTLA-4 monoclonal antibody (mAb) may lead to increased expansion of vaccine-induced T cells. (2) hTERT-specific T cells enter circulation and (3) infiltrate the tumor. Normalization of the tumor vasculature through inhibition of VEGF may facilitate an increased influx of T cells. (4) T cells recognize hTERT on local antigen-presenting cells in the context of an MHC class II molecule and directly stimulate local CD8+ T cells through IL-2 secretion and indirectly through co-stimulation of DCs (CD40L-CD40 interaction), leading to enhanced cross-presentation 44. (5) MHC class II expressing tumor cells can be directly killed through cytokine secretion or indirectly through activation of CD8+ cells and macrophages (M φ) (44, 48). Anti-PD-1/L1 mAb may provide increased effector activity of vaccine-induced T cells in the tumor by blocking regulatory signals on T cells (PD-1) or tumor cells (PD-L1). (6) Lysed tumor cells release hTERT or mutated peptides, which in turn are (7) phagocytosed by DCs and presented to T cells providing either intra- or intermolecular epitope spreading and broadening of the anti-tumor immune response (52). Anti-CTLA-4 mAb may, in turn, support further priming and expansion of anti-tumor T cells. Figure created with BioRender.com.

CD8+ cytotoxic T lymphocytes were long considered the most potent anti-tumor effectors in the adaptive immune system, but lately, the focus has shifted towards the importance of CD4+ T helper lymphocytes as an opportunity to achieve tumor recognition and T cell infiltration in an immunosuppressive TME. CD8+ T cells have the ability to directly kill cancer cells expressing their cognate antigen in the context of an HLA class I molecule. For this interaction to occur, the antigen must be processed internally by the cancer cell in a multi-step process to be loaded onto HLA class I molecules (40). CD4+ T cells are, on the other hand, activated through interaction with their antigen in the context of an HLA class II molecule, typically expressed by antigen-presenting cells (APCs), but also upregulated on cancer cells by IFN-γ stimulation and thus frequently expressed by immunogenic tumors (41). Activated CD4+ T cells orchestrate an immune response through the release of pro-inflammatory cytokines. As reviewed elsewhere (42–44), critical features of CD4+ T helper 1 (Th1) cells in anti-tumor immunity include induction of effective antigen presentation by APCs, augmentation of CD8+ T cell responses, T cell homing to the tumor (45), direct and indirect tumor cell killing (46–48), and formation of memory T cells (49, 50). The multifaceted functions of Th1 cells may thus support virtually all steps in the cancer immunity cycle (51) (**Figure 2**). As hTERT is expressed throughout the tumorigenesis, this CD4+ Th1 response may stay activated and relevant regardless of the tumor’s rapidly evolving genetic makeup, providing the immune system with an opportunity to mount an individually tailored immune response to relevant target antigens. This concept of an “*in vivo* personalized vaccine” stands as an alternative strategy to “*ex vivo* personalized vaccines” where the selection of tumor targets is based on predicted HLA class I binding neoantigens.

A caveat of the CD4+ immune response is the different subtypes that are considered good or bad with respect to anti-tumor immunity. The Th1 phenotype is typically considered ideal, and T(reg) (CD4+, FOXP3+) is considered immunosuppressive (44). The roles of other phenotypes, such as Th17 and Th2, in cancer immunity, are not as well established (53). The differentiation into the Th subsets relies on the priming environment (54), and as such, the phenotype may be affected by vaccine design, vaccine administration route, and use of an adjuvant. Furthermore, Hansen et al. found that samples from the CTN-2000 trial, where patients received hTERT vaccination as monotherapy, displayed a more Th1-polarized phenotype than samples from the CTN-2006 trial evaluating the same vaccine, GV1001, as maintenance treatment after chemoradiotherapy (55). This indicates that disease stage and previous therapies may also affect the phenotype of vaccine-induced T cells and thus anti-tumor efficacy.

#### Telomerase-Based TCV Platforms

The most frequently utilized vaccination platform for telomerase-based TCVs is peptide vaccines (23/34 clinical trials). Peptide vaccines aim to elicit an adaptive immune response by *in vivo* uptake of the peptides by APCs at the vaccination site and subsequent presentation of embedded epitopes to naïve T cells leading to their expansion. Peptides are probably the preferred platform owing to their relatively long shelf-life, simple synthesis and administration route, requiring only intradermal or subcutaneous injection along with a vaccine adjuvant. The skin serves as an ideal administration route, as it contains a dense population of various dendritic cell subsets (56). The first TCVs developed commonly consisted of short peptides (up to 10 amino acids) as they can be loaded directly onto HLA class I molecules and induce CD8+ immune responses (57). Recently, however, synthetic long peptides (SLPs) have been in focus since they have the potential to provide cross-presentation by APCs leading to both class I and II presentation, and hence CD8+ and CD4+ immune responses, respectively (58, 59). The use of epitope dense SLPs also allows enrollment of patients independently of their HLA types (such as with GV1001 and UV1), whereas many short peptide vaccines have been tailored to fit single HLA class I molecules, thus limiting inclusion to patients harboring this HLA type (39) (**Table 1**).

**Table 1:** hTERT TCV candidates evaluated in clinical trials covering various indications over the past two decades (autologous cell-based therapies are not included).

Drug name	Adjuvant	HLA screening	Indications tested	Combinations	Highest development stage	Active trials
**Peptide vaccines**						
GV1001	GM-CSF	No	- Melanoma (60, 61) - Pancreatic cancer (1, 62) - NSCLC (63, 64) - Hepatocellular carcinoma (65)	- Temozolomide - Cyclophosphamide - Gemcitabine	Phase III	None in cancer
hTERT:540-548 peptide	Montanide	HLA-A*0201	- Metastatic cancer (66)		Phase I	No
Vx-001	Montanide	HLA-A*0201	- NSCLC (67, 68) - Advanced solid tumors (69, 70)		Phase II	No
GX301	Montanide and imiquimod	HLA-A2	- Advanced prostate or renal cancer (71)		Phase I/II	
UV1	GM-CSF	No	- Prostate cancer (72) - NSCLC (73) - Melanoma (74)	- Ipilimumab	Phase II	Yes
UCPvax*	Montanide					Yes
**RNA-based vaccines**						
mRNA vaccine	GM-CSF	No	- Advanced renal cancer (75)		Phase I/II	No
**DNA-based vaccines**						
INVAC-1	No	No	- Various solid tumors (76)		Phase I	Yes
INO-5401*						Yes

Clinical trial reports available on Pubmed are referenced under indications tested. *There are currently no reports from clinical trials evaluating UCPvax and INO-5401 on Pubmed.

Other vaccine platforms include autologous dendritic cell (DC) vaccines, utilized in 10/34 clinical trials, ensuring *in vitro* antigen loading in DCs for presentation to T cells *in vivo.* This platform necessitates complex logistics and competence, including patient leukapheresis and subsequent DC cell culture and antigen pulsing, after which the treated DCs are transfused back to the patient (77). An mRNA vaccine has also emerged in the hTERT TCV pipeline, coding for several TAAs (MUC1, CEA, Her-2/neu, telomerase, survivin, and MAGE-A1), showing induction of CD4+ and CD8+ immune responses in a phase I/II trial (75). The last platform evaluated in clinical trials is DNA vaccines. Two hTERT-based vaccines utilize this platform, INVAC-1 and INO-5401. INVAC-1 consists of a plasmid DNA that encodes a modified inactive version of hTERT and has been shown to induce CD4+ and CD8+ immune responses in a phase I study (76). The advantage of a DNA or RNA-based vaccine is the incorporation of the whole gene. Still, the possible lack of adjuvant capability of DNA vaccines, in general, remains the biggest bottleneck for future clinical efficacy (78, 79). Results from studies with INO-5401 are not yet published in peer-reviewed journals. Dillard et al. recently described a novel telomerase immunotherapy platform, hTERT-targeting T cell receptor (TCR) therapy, showing both *in vitro* and *in vivo* anti-tumor efficacy in an animal model (80).

#### Vaccine Adjuvants

Peptide vaccines require an adjuvant to elicit an immune response, as naked peptides by themselves are poorly immunogenic. There are primarily two adjuvants currently employed in telomerase TCV trials. Granulocyte-macrophage colony stimulating factor (GM-CSF) is the most frequent, being utilized in 12/34 clinical trials, and incomplete Freund’s adjuvant (IFA)(Montanide ISA-51) being the second most common (11/34). Recombinant human GM-CSF acts to recruit APCs to the vaccination site and initiate differentiation and activation (81). Importantly, GM-CSF is water-soluble as opposed to IFA, which is a water-in-oil emulsion. As described by Hailemichael et al., IFA creates a depot effect at the vaccination site, which may sequester antigen-specific T cells and thus prevent migration of T cells to the tumor site (82), possibly explaining the failure of the gp100 vaccine (with IFA adjuvant) in the landmark study of ipilimumab and gp100 combination vs. ipilimumab alone or gp100 alone (2). Thus, GM-CSF or other water-soluble adjuvants may be the most suitable vaccine adjuvant for peptide cancer vaccines, but likely requires repeated administrations to compensate for the lack of depot effect. GM-CSF can also display dual roles as a proinflammatory signaling moiety in the immune system, impacting immunological responses depending on the cell type expanded in response to GM-CSF stimulation (83). As a drug, the dose of GM-CSF and possibly the formulation of GM-CSF may also impact the pharmacodynamic response. Nevertheless, hTERT-targeting TCVs with both GM-CSF and IFA adjuvants have been shown to induce immune responses in blood with no apparent differences in frequency.

Recently, compounds that conjugate an adjuvant directly with the vaccine peptides have garnered interest and hold promise due to their efficiency in providing targeted adjuvant effect (84, 85). Such compounds have, however, not been evaluated with telomerase peptides.

#### Monitoring of Immune Responses in Clinical Trials

Inherent to the potential clinical benefit of a therapeutic cancer vaccine is its ability to induce immune responses, and several clinical trials with telomerase vaccines have shown a positive correlation between vaccine-induced immune responses and survival (60, 62, 63). The rate of immune responders has varied across the clinical trials, ranging from zero percent in a trial in hepatocellular carcinoma (65) to 78 percent in a trial in melanoma (60) with the most frequently evaluated hTERT TCV, GV1001. The discrepancy observed across trials may have several causes other than suboptimal vaccine design. The fraction of vaccine-specific T cells in circulation is likely low since T cells primarily reside in lymphoid tissues (or the tumor) and are thus not captured by drawing <100 ml of peripheral blood (86, 87). Thus, ideal methodologies to monitor immune responses have yet to be developed. Regardless, the relatively high immune response rates observed across different hTERT trials suggest that central tolerance is not imposed on hTERT-specific T cells and that vaccination may induce high-affinity T cell responses. Furthermore, longitudinal immune monitoring with the proliferation assay has demonstrated persisting immune responses detectable up to 7 years after initial vaccination and serve as evidence of induction of immune memory with lasting proliferative potential (60, 63). Interestingly, late peaks in immune responses have coincided with clinical events, suggesting a natural boosting of the immune response by recurring tumors (74).

#### Safety

The most reported side effect with telomerase-targeting cancer vaccines is injection site reactions, including local erythema and pruritus, and flu-like symptoms such as fever, muscle and joint pain, and fatigue. Generally, telomerase vaccination appears to be well tolerated, with only a few cases of serious adverse events reported (39). As an endogenous self-antigen, an immune response against hTERT is associated with a theoretical risk for off-tumor on-target toxicity. However, due to the physiologic function of hTERT, its expression pattern is limited to specific sites of highly proliferating cells and stem cells such as bone marrow, testis, embryo, and placenta (25, 26). These tissues are described as specific immune-privileged sites due to their local tolerogenic environment (88–93). Immune tolerance mechanisms are put in place at these tissues to protect from immunological insults, which would potentially be deleterious given the critical physiologic functions of these cells. These factors are likely also preventing autoimmunity resulting from off-tumor on-target reactions from vaccine-induced T cells, further substantiated by the lack of immune-related adverse events observed in hTERT-expressing tissues in clinical trials with various telomerase vaccines. To explore the theoretical risk of inducing immune responses against stem cells, bone marrow histological examinations have been performed in a subset of clinical trials with telomerase peptides or DC vaccines, showing no discernable changes after vaccination (64, 94, 95). Although the reports from conducted clinical trials indicate a tolerable safety profile, novel compounds utilizing different vaccine platforms and treatment combinations may elicit more potent immune responses, and consequently, increase the risk for off-tumor effects. Checkpoint inhibitor combinations are especially relevant in this context, as their physiologic function is to limit autoimmunity, and their inhibition may thus lower the threshold for such off-tumor reactions. A clinical trial of an hTERT vaccine in combination with the checkpoint inhibitor ipilimumab did not report a change in side effect panorama in treated patients (n=12) (74). However, larger studies are needed to conclude on the safety of combining checkpoint inhibitors and telomerase vaccines.

## Considerations for Optimizing the Therapeutic Potential of Telomerase-Targeting Vaccines

Considering that most tumors rely on hTERT, telomerase appears to be an almost universal cancer antigen, and hTERT targeting TCVs are therefore potentially broadly applicable, reflected in the extensive set of indications tested (see **Table 1** below) (39). Still, to optimize the therapeutic potential of hTERT-vaccination, the selection of indications should be made based on tumor hTERT expression and factors that may restrict the intratumoral activity of T cells.

### Telomerase Activation and Expression

#### Tumor hTERT Promoter Mutations

Mechanisms of hTERT gene activation in cancer have been widely studied, and somatic mutations in hTERT are the most well described. Somatic mutations in the coding region of hTERT appear to be rare, but mutations in the promoter region are common (appx. 19% of tumors) (96). Furthermore, two recurrent, mutually exclusive, hTERT promoter mutations (C228T and C250T, collectively referred to as hTERTp) have been found in 71% of melanomas and confer a 2-4-fold increase in hTERT promoter transcriptional activity (97). These two mutations have the same functional consequence, creating an hTERT promoter binding site (GGAA) for ETS (E-twenty-six) transcription factors. These mutations have also been significantly associated with BRAF mutations in melanoma (96–98). BRAF mutations lead to elevated ETS transcription factors, which may bind to the hTERT promoter binding site resulting from the promoter mutations, thereby working in concert to increase hTERT activity (99).

As reported by others (36, 97, 98), hTERTp appears to be more frequent in metastatic than in primary melanoma tumors. These findings suggest that hTERTp is either a late event or confer a survival advantage for the cancer clone, leading to its higher frequency in advanced disease. In contrast, in another study of 58 matching primary and metastatic melanoma lesions, hTERTp appeared more frequently in primary tumors and were exclusively observed in primary or metastatic lesions in 17% and 7% of cases, respectively (37). Additionally, a clonal distribution of hTERT promoter mutations has been reported in early-stage hepatocellular carcinoma (100). These discordant observations indicate variations in the phylogeny of promoter mutations across tumors and possibly indicate different roles of hTERTp in the tumorigenesis of the individual tumor. The heterogeneity of hTERTp could indicate an intratumoral heterogeneity of hTERT expression. In line with this concept, there are variations in the hTERT expression levels both within and across cancer cell lines (101). This observation may be due to cell-level variation in the transcription of hTERT occurring at different phases of the cell cycle (102), giving rise to variations at a specific moment in time and a continuous fluctuation with longitudinal observations. However, the regulation of hTERT expression is convoluted and a contended area of research (103). Notably, these promoter mutations were not found in benign lesions, kidney cancers, pheochromocytomas, or gastrointestinal stromal tumors (96, 104).

#### Copy Number Alterations

Another possible mechanism of hTERT activation is copy number amplification (CNA) of the hTERT gene. hTERT CNA is over-represented in melanomas (105) and also described with a relatively high frequency (30%) in lung cancer, breast cancer, cervical carcinomas, and neuroblastoma (106). Another study of 2,210 solid tumors found that CNA at chromosome 5p, where the hTERT gene is located, was the eighth most common chromosomal gain (13.2%) (107). Furthermore, hTERT CNA is associated with increased hTERT expression among cancer cell lines and primary solid tumors (106).

#### Epigenetic Changes Affecting Telomerase Expression

Genetic, as well as epigenetic changes, can affect telomerase activity. Epigenetic changes are not linked to changes in the nucleotide sequence, but are still preserved with cell division, impacting gene activity and expression. Commonly, the impact on gene regulation *via* epigenetic changes is controlled by specific methylation patterns or histone modifications (acetylation), often at non-coding sites such as the promoter region. In the case of telomerase, there are two different regions in the promoter that affect expression, one of which is a non-methylated region in the proximal region of the promoter, which, in its non-methylated state, is associated with active telomerase expression. However, it has also been discovered that there is a region called the *TERT* Hypermethylated Oncological Region (THOR) (108–112). The THOR is unusual in its behavior, as it is repressing expression in its unmethylated state, and as such, it is related to hTERT activation and cancer progression upon hypermethylation. Interestingly, THOR hypermethylation is more common in cancers known to have a low frequency of hTERTp (prostate, lung, colon, and breast cancer), indicating THOR hypermethylation as an alternative hTERT activation mechanism for these tumor types. Mutations in the promoter region can also affect the hypermethylation pattern (113) and possibly give rise to synergistic effects between the genetic and epigenetic changes when it comes to hTERT expression.

#### Alternative Lengthening of Telomeres

Some tumors utilize another mechanism of telomere maintenance than hTERT, termed alternative lengthening of telomeres (ALT), documented in <5% of all cancers (114). This mechanism is important as tumors that harbor the ALT phenotype would likely not benefit from hTERT vaccination. ALT appears to be more regularly employed in non-epithelial cancers such as sarcomas and brain tumors, and mutations in the telomere binding proteins ATRX and DAXX have been described to induce the ALT phenotype (115). hTERT promoter and ATRX mutations are mutually exclusive, indicating that hTERT activation and ALT do not co-occur in the same tumor (104).

Although these findings indicate hTERT and ALT being two distinct telomere maintenance pathways, the transfection of hTERT into an ALT-utilizing cell line has shown that these two mechanisms can run in parallel. However, when a telomerase-positive cell line is fused with an ALT-utilizing cell line, ALT is repressed, indicating that a factor other than hTERT represses ALT in non-transfected models (116). The possibility of ALT activation as a mechanism of resistance from hTERT inhibition has been evaluated in cancer cell line models, and they did indeed show that cells surviving the telomere crisis after telomerase inhibition could elongate the telomeres in an ALT-like manner. However, resistant, telomerase-negative cancer cells were significantly less invasive and tumorigenic (117, 118). These findings are in line with the described tumorigenic contribution of hTERT besides telomere elongation (119).

Although the direct inhibition of hTERT may lead to resistance through activation of the ALT pathway, the immune pressure imposed on tumors through vaccination likely avoids this type of resistance mechanism. CD4+ T cells are activated upon interaction with its cognate antigen on HLA class II expressing cells, such as APCs. APCs scavenge the TME and phagocytose remnants of dying cancer cells. They present this content to CD4+ T cells, which in turn release inflammatory cytokines and stimulate other immune cells (120). Thus, this indirect and dynamic approach to enhance anti-tumor immune responses likely circumvents tumor resistance by mechanisms typically seen with direct inhibition, such as tyrosine kinase inhibitors. More plausible resistance mechanisms are likely similar to those of acquired resistance to CPIs, characterized by evasion from the immune system through disruption of shared pathways of immune activation (e.g., tumor loss of HLA, B2M, and IFN-γ signaling) (121).

### Immune Permissive Tumor Microenvironment

The anti-tumor effect of vaccine-induced T cells likely relies on their potential to home to the tumor or tumor-draining lymph node. Multiple mechanisms are exploited by the tumor to restrict the infiltrative potential of T cells (122) and could thus limit the anti-tumor efficacy of TCVs. Tumors can be characterized into three broad categories based on the presence of tumor-infiltrating lymphocytes (TILs) and immunosuppressive factors in the TME, or the so-called immunophenotype (123). These categories include the inflamed, excluded, and desert phenotypes. Inflamed tumors have infiltration and activation of immune cells, marked by elevated PD-L1 expression and IFN-γ signaling, and they typically respond well to CPI therapy. Immune excluded tumors have an abundance of immunosuppressive factors such as TGF-β and MDSCs. Both the inflamed and excluded phenotypes can harbor TILs, but at a much lower level in the excluded phenotype. The immune desert phenotype has few TILs and is characterized by elevated WNT/β-catenin signaling and fatty acid metabolism.

PD-L1 expression and IFN-γ signaling provide evidence of infiltration and activation of T cells and indicate an immune permissive TME. However, a lack of these features may not accurately determine the T cells’ infiltrative potential in these tumors, as evidenced by responses to CPIs in TMB high and PD-L1 low tumors. Thus, no accurate predictive marker exists to determine an immune permissive TME, and variations between the inflamed, excluded, and desert immunophenotypes exist both within and across tumors, making the selection of exclusively permissive tumor types difficult (124). Lastly, the tumor consists of a dynamic environment that may be affected by TCV-induced T cells overcoming immunosuppression *via* intratumoral activation of CD4 T cells and secretion of pro-inflammatory cytokines. However, tumor types where CPIs have demonstrated clinical responses may be the best and easiest guide for selecting immune permissive tumors.

### Immunologically Rational Treatment Combinations

Although telomerase vaccines have proven to induce immune responses in blood, the T cell population expanded through vaccination is likely constrained by intrinsic and tumor-induced regulatory mechanisms, such as the checkpoint molecules CTLA-4 and PD-1/L1, respectively, and a varying degree of immunosuppression within the TME (depending on immunophenotype). Previous late-phase TCV trials may have failed due to the lack of appropriate treatment combinations addressing these regulatory mechanisms or the immunosuppressive milieu of the tumor. Therefore, it is necessary to leverage the recent advancements in immunotherapy and combine TCVs with checkpoint inhibitors or other therapeutic molecules modulating the TME in favor of T cell expansion, infiltration, and effector function.

The CTLA-4 checkpoint primarily acts to regulate the expansion of activated T cells by competitive inhibition of the binding of CD28 on the T cells with B7 ligands on the APCs, thereby disrupting co-stimulation of primed T cells (125). In the TCV setting, the systemic administration of an anti-CTLA-4 monoclonal antibody may provide not only enhanced expansion of spontaneously primed T cells in the tumor-draining lymph node, but also vaccine-induced T cells in the lymph node draining the vaccination site. Thus, the combination of anti-CTLA-4 and a TCV may allow increased expansion of vaccine-induced T cells after priming, addressing a central challenge when targeting TAAs. Only one completed clinical trial has combined the anti-CTLA-4 monoclonal antibody ipilimumab with a telomerase TCV (NCT02275416). Results from this trial evaluating UV1 combined with ipilimumab in advanced melanoma were presented at ASCO-SITC 2020 and showed early induction of immune responses in 10/11 (91%) of the evaluable patients (74).

The PD-1 immune checkpoint is upregulated on T cells upon activation, and its ligand PD-L1/L2 is upregulated on tumor cells in response to inflammatory cytokines (IFN-γ). This axis thus serves to restrict the effector capacity of T cells within the tumor by promoting T cell anergy and exhaustion (125). By blocking this interaction, the vaccine-induced T cells may achieve greater effector activity and tumor cell killing. The anti-tumor synergy of vaccination and dual checkpoint blockade has previously been demonstrated in animal models (126–128).

There are at least nine ongoing clinical trials investigating hTERT vaccines with an anti-PD-1/L1 monoclonal antibody (**Table 2**). The telomerase peptide vaccine UV1 is combined with pembrolizumab (anti-PD-1) in a phase I clinical trial (NCT03538314) and with nivolumab (anti-PD-1) and ipilimumab in two randomized phase II clinical trials in malignant melanoma (NCT04382664) and mesothelioma (NCT04300244), respectively. Two more studies that have yet to begin patient recruitment are investigating UV1 in combination with durvalumab (anti-PD-L1) and olaparib (PARP inhibitor) in relapsed ovarian cancer and with pembrolizumab in head and neck cancer. The peptide vaccine UCPVax is combined with nivolumab in a randomized phase II clinical trial (NCT04263051) and with atezolizumab (anti-PD-L1) in HPV+ cancers (NCT03946358). INO-5401, a DNA vaccine targeting Wilms tumor gene-1 (WT1), prostate-specific membrane antigen (PSMA), and hTERT, is evaluated in two phase I/IIa studies, in combination with atezolizumab in a urothelial carcinoma study (NCT03502785) and in combination with cemiplimab (anti-PD-L1) in newly diagnosed glioblastoma (NCT03491683), respectively. Although the combination with anti-CTLA-4 and anti-PD-1/L1 may be superior in expanding vaccine-induced T cells, this combination also poses a severe toxicity profile. Therefore, adding a TCV must not significantly exacerbate toxicity to achieve a feasible risk/benefit profile for the combination treatment.

Treatment with inhibitors of vascular endothelial growth factor (VEGF) can normalize the vasculature to allow increased infiltration of T cells, synergizing with adoptive cell transfer (129), and conceivably also TCVs (130). Furthermore, the immunosuppressive cytokine TGF-β is involved in modulating the immune excluded TME and can drive the differentiation of primed CD4+ T cells to the immunosuppressive T(reg) subtype (131–133). Recently, TGF- β has been identified to selectively suppress CD4+ Th2 cells (134). Targeting these suppressive TME factors, or so-called cancer environment immunotherapy, may be a novel approach that synergizes with TCVs (135–137).

## Telomerase-Based TCVs in Clinical Development

Several anti-telomerase vaccine candidates have been evaluated in clinical trials during the last two decades (**Table 1**). Opportunities for scientifically rational treatment combinations (e.g., checkpoint inhibitors) have provided renewed interest in TCVs targeting telomerase with 13 ongoing trials (**Table 2**). Novel vaccine platforms have also emerged in the hTERT TCV pipeline to include DNA and RNA-based vaccines. TCVs that are either in active development or have been evaluated in more than one trial are described in more detail below.

**Table 2:** Several hTERT targeting TCVs are currently evaluated in active clinical trials and are often combined with different checkpoint inhibitors.

Vaccine	Indication	Combination	Phase (NCT)
UV1	Advanced melanoma	Pembrolizumab	I (NCT03538314)
	1^st^ line treatment of advanced melanoma	Ipilimumab and nivolumab	II (NCT04382664)
	2^nd^ line treatment of unresectable malignant pleural mesothelioma	Ipilimumab and nivolumab	II (NCT04300244)
	Maintenance treatment for relapsed ovarian cancer	Durvalumab and olaparib	II (NCT04742075)
	Head and neck cancer	Pembrolizumab	II
UCPvax	2^nd^ line treatment of advanced NSCLC	Nivolumab	II (NCT04263051)
	Pre-treated advanced NSCLC		I/II (NCT02818426)
	Pre-treated glioblastoma	Vaccination starts at >1 month after radiochemotherapy	I/II (NCT04280848)
	Pre-treated locally advanced or metastatic HPV+ cancers	Atezolizumab	II (NCT03946358)
INVAC-1	Various solid tumors (exploratory addendum)		I (NCT02301754)
INO-5401	BRCA ½ mutation carriers, with or without cancer		I (NCT04367675)
	Locally advanced or metastatic urothelial carcinoma	INO-9012 and atezolizumab	I/II (NCT03502785)
	Newly diagnosed glioblastoma	INO-9012 and cemiplimab	I/II (NCT03491683)

### GV1001

The only hTERT-based vaccine with market approval is the telomerase vaccine is GV1001, a 16-mer peptide covering the active site of hTERT. GV1001 was also one of the first telomerase-based TCVs evaluated in the clinic and has been assessed in 7 clinical trials, covering pancreatic cancer, melanoma, NSCLC, and hepatocellular carcinoma. GV1001 vaccination encompasses intradermal injection of 0.56 mg of vaccine peptide and 75μg of GM-CSF as an adjuvant. The treatment schedule consists of 3 vaccinations during week 1, and one vaccination on weeks 2, 3, 4, and 6, and monthly vaccinations thereafter.

GV1001 was evaluated in the only phase III trial with hTERT-targeting TCVs to date, assessing chemotherapy with or without GV1001 in patients with locally advanced or metastatic pancreatic cancer (1). The study did not meet its primary endpoint of improved overall survival with the addition of GV1001 to chemotherapy. There are several possible causes for the failure to meet the primary endpoint in this trial. First, the immune response rate was substantially lower than expected, at 38%. This low rate essentially reduces the population eligible for a clinical effect of vaccination by almost 2/3. Second, as previously argued, immunologically rational treatment combinations are likely necessary to bring out the true clinical potential of TCVs, and even though chemotherapy may induce immunogenic cancer cell death, it is uncertain which effect it has on a vaccine’s ability to induce appropriate T cells responses. Third, pancreatic cancer is known to be desmoplastic with a high concentration of cancer-associated fibroblasts inducing an immunosuppressive TME, limiting the potential for immunotherapy (138), as supported by a lack of efficacy of anti-PD-L1 in pancreatic cancer (139). hTERT expression in pancreatic cancer is also relatively low compared to other cancer types (**Figure 1**). Although the study failed to achieve an OS benefit in the intention-to-treat population, retrospective subgroup analysis showed that eotaxin levels predicted the benefit of the addition of GV1001 to chemotherapy (140). Based on these data, GV1001 received conditional market approval in Korea for patients with locally advanced or metastatic pancreatic cancer and an elevated serum eotaxin level. Eotaxins function as chemoattractants for immune cells and could thus enhance infiltration of T cells to the tumor. However, the mechanistic link and its predictive value need confirmation in future studies.

### UV1

In the CTN-2000 trial, which evaluated GV1001 in NSCLC (n=26) (63), patients who mounted a vaccine-specific immune response had an improved overall survival (OS) compared to non-immune responders (median 19 months vs. 3.5 months; *P* < 0.001). Inderberg et al. subsequently characterized the immune response in long vs. short-term survivors to elucidate immunological mechanisms characteristic for patients with a clinical benefit (52). They evaluated immune responses in patients using a library of overlapping peptides, 24 15-mers and one 30-mer, covering the active site of hTERT. Long-term surviving immune responders demonstrated so-called intramolecular epitope spreading, i.e., induction of *de novo* immune responses against other, structurally unrelated, epitopes within the hTERT molecule. Moreover, immune responses against specific hTERT peptides were correlated with survival benefit and were not detected in short-term survivors. Resultantly, three highly immunogenic epitope dense peptides with broad HLA-coverage associated with improved survival were selected for a next-generation telomerase vaccine, UV1. These peptides were relatively long (two 15-mers and one 30-mer), requiring intracellular processing by the APCs, allowing individual selection of epitopes matching patient-specific HLA-alleles, thereby ensuring wide population coverage and CD8+ and CD4+ immune responses. The UV1 vaccine is administered intradermally at 300 μg of peptides with 75 μg GM-CSF as an adjuvant. The treatment schedule consists of 3 vaccinations during week 1 and up to 5 booster vaccinations thereafter.

### INVAC-1

INVAC-1 is a DNA-based vaccine containing a modified and enzymatically inert hTERT gene variant that can be administered by intradermal injection and give rise to hTERT protein exposure in vaccinated individuals. The vaccine has been modified to improve protein degradation through a ubiquitin sequence introduction. In a recent publication presenting the phase I safety readout, the vaccine was found safe with no dose-limiting toxicity with vaccine-induced T cell expansion established (76).

### VX-001

VX-001 is a peptide-based telomerase vaccine containing two 9 amino-acid peptides (one wild-type sequence and one mutated/optimized sequence) that aim to expand cytotoxic hTERT specific T cells in an HLA-A2 selected population. The peptides are formulated in Montanide and at low non-physiological pH. In a phase 2 study, the first and second vaccination was performed using the mutated/optimized sequence, and the third vaccination and onwards (four additional vaccinations) were performed using the wild type sequence. In patients with a tumor under control, vaccination was continued every 3 months. Despite the failure of meeting the primary endpoint in the non-selected cohort, there was a significantly improved overall survival in immune responders, and this was also established in immune responders with an otherwise unfavorable prognosis based on elevated LDH and γGT (141). This may indicate that the vaccine provided an effect and that the improved survival in immune responders was not only a result of a selection of patients that would survive longer regardless of treatment.

## Future Perspectives

As the tumor develops, so do its mechanisms for evading the immune system, necessitating strategic treatment combinations to overcome tumor intrinsic or extrinsic immune escape mechanisms in the advanced disease setting. To fully harvest the potential of synergy by combinatorial treatment strategies, there is a need to understand the kinetics of each component to align for optimal efficacy. Traditionally, treatments are given simultaneously to impose the greatest pressure on the tumor. However, with immunotherapeutic approaches, there could be additional gains achieved by appropriate timing, as demonstrated for sequential administration of a tumor cell vaccine and anti-CTLA mAb (142) and concurrent administration of anti-PD-1 and vaccination (143). A further understanding of how to best combine various immunotherapies will be essential in future testing. To date, most hTERT-targeting TCVs have been evaluated in advanced disease and in heavily pre-treated patients. As hTERT is a relevant antigen along the cancer disease continuum, these TCVs could be employed in earlier disease settings, perhaps with reduced reliance on treatment combinations. Indeed, there are studies showing promising results of monotherapy TCVs in patients with low tumor burden (144–147).

No clinical trials have yet incorporated relevant target biomarkers, such as tumor hTERT expression. Such biomarkers could potentially allow a narrower selection of patients eligible for effect from vaccination and thus improve on clinical efficacy. Another interesting approach is the application of hTERT-targeting TCVs in TMB-low tumors, where CPIs show limited effect and other TCV strategies such as neoantigen vaccines are less relevant. Biomarkers of possible resistance should also be considered for future studies. Such predictive biomarkers can include tumor loss of function mutations in HLA, B2M, and IFN-γ signaling.

## Conclusion

Although telomerase vaccines have been under investigation for almost two decades, recent studies elucidating the mechanisms behind the lack of effect from CPIs provide renewed enthusiasm for TCVs, in general, as a means to improve clinical outcomes. Telomerase as a TCV target has apparent advantages due to its universal presence and essential function in almost all cancer types, providing spatiotemporal relevance to the induced immune response and limiting possible escape mechanisms for the tumor.

TCVs should robustly elicit both the CD4+ and CD8+ compartments of the adaptive immune system for optimal intratumoral activity of the induced immune response. Higher tumor expression of telomerase is likely to confer a heightened anti-tumor immune response in vaccinated patients. Several factors are involved in regulating telomerase expression, with hTERT promoter mutations being the most well described. Furthermore, BRAF mutations have been shown to act synergistically with hTERT promoter mutations to increase telomerase activity. Considering the high frequency of hTERT promoter mutations, BRAF mutations, and copy number amplification of the hTERT gene in melanoma, patients with this cancer are more likely to achieve benefit from vaccination.

Immunologically rational combinations, such as anti-CTLA-4 and anti-PD-1/L1, are likely necessary to bring out the true clinical potential of hTERT-targeting TCVs. There are already several phase II randomized controlled trials evaluating hTERT targeting TCVs in combination with CPIs with anticipated read-outs. The tumor type targeted should be assessed for its microenvironment as multiple factors, such as TGF-β, are to a varying degree contributing to local immunosuppression across tumor types. Although highly immunosuppressive tumors are likely to be more challenging to target, novel compounds addressing these tumor environmental factors are emerging and could possibly provide synergistic effects with vaccination.

Development of better methodologies to evaluate immune responses in patients is needed and should provide a more comprehensive quantification of the induced immune responses and insights into the optimal phenotype of the T cells. Additional translational studies on the intratumoral activity of the induced T cells would strengthen the rationale for further development of hTERT targeting TCVs.

## Author Contributions

EBE and SM have reviewed the field. EH and GG have provided guidance on relevant considerations with respect to tumor biology and anti-tumor immunity. All authors contributed to the article and approved the submitted version.

## Funding

The Norwegian Research Councils grant number is 298864 and Ultimovacs ASA fund the Ph.D. project for the corresponding author. The funder was not involved in the study design, collection, analysis, interpretation of data, the writing of this article or the decision to submit it for publication. The University of Oslo funds the article processing fee.

## Conflict of Interest

EBE and GG are employees of Ultimovacs ASA. SM and GG are shareholders in Ultimovacs ASA. SM is a founder, shareholder, and board member of Immuneed AB, Vivologica AB, and Strike pharma AB and an employee of Ultimovacs AB. GG is an inventor of a UV1 vaccine patent.

The remaining author declares that the research was conducted in the absence of any commercial or financial relationships that could be construed as a potential conflict of interest.
